# Target discovery for T cell therapy: next steps to advance Immunotherapies

**DOI:** 10.1186/s40425-015-0061-5

**Published:** 2015-07-21

**Authors:** Adrian Bot, Joanna E Brewer, Zelig Eshhar, Stanley R Frankel, Emma Hickman, Achim A Jungbluth, Richard Morgan, Yoav Peretz, Laszlo Radvanyi, Carlos A Ramos, Paul F Robbins, Kai W Wucherpfennig

**Affiliations:** Translational Medicine, Kite Pharma Inc., 2225 Colorado Avenue, 90404 Santa Monica, CA USA; Cellular Biology, Adaptimmune Ltd., Abingdon, UK; Immunology, The Weizmann Institute of Science, Rehovot, Israel; Oncology Early Development, Therapeutic Area Head, Amgen, Thousand Oaks, CA USA; Target Validation, Immunocore, Abingdon, UK; Immunohistochemistry, Pathology, Memorial Sloan-Kettering Cancer Center, New York, NY USA; Immunotherapy, Bluebird Bio, Boston, MA USA; Immunology, ImmuneCarta, Caprion, Montreal, Quebec Canada; Lion Biotechnologies, Woodland Hills, CA USA; Medicine, Section of Hematology-Oncology, Baylor College of Medicine, Houston, TX USA; DNA Sequencing and FACS Cores, Surgery Branch; Staff Scientist, Center for Cancer Research, National Cancer Institute, Bethesda, MD USA; Cancer Immunology & AIDS, Dana-Farber Cancer Institute; Professor of Neurology at Harvard Medical School, Cambridge, MA USA

**Keywords:** Immunotherapy, Adoptive T cell therapy, Chimeric antigen receptors, T cell receptors, Tumor infiltrating lymphocytes, Targets, Adoptive T cell therapy, Antibodies, Vaccines

## Abstract

Investigators from academia and industry gathered on August 14, 2014, in Boston at the Inaugural ImVacS conference entitled “Target Discovery for T Cell Therapy: Next Step to Advance Immunotherapies”. Novel targets, discovery strategies and enabling technologies were presented and discussed.

T cell target discovery remains one of the biggest challenges facing the immunotherapy field. With treatments becoming ready for prime-time, researchers need to increase their discovery efforts to meet patient needs and continue advancing the field. While there is a consensus that current targets considered for adoptive T cell therapies are few and challenging, there is an emerging realization that novel enabling technologies must also be developed, to afford more potent and safer T cell products against novel targets.

Cambridge Healthtech Institute’s Inaugural Target Discovery for T Cell Therapy gathered leaders from academia and industry actively engaged in discovery and translation of novel targets for T cell immunotherapy: http://www.imvacs.com/t-cell. Experts shared strategies for chimeric antigen receptors (CARs), T cell receptors (TCRs), tumor infiltrating lymphocytes (TILs), and novel methods for novel targets (Figure 1). Clinical data were showcased as well as in depth examination of where the field is headed.

**Figure 1:**
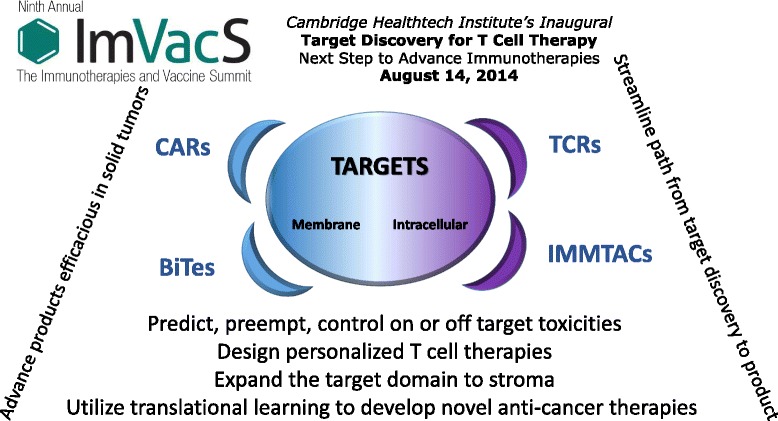
Major themes explored at the Cambridge Healthtech Institute’s Inaugural Event titled “Target Discovery for T Cell Therapy: Next Step to Advance Immunotherapies”.

The event was organized in four sessions: the first two covered current Chimeric antigen receptor (CAR) and T cell receptor (TCR) technologies and associated targets that have been explored during the last few years, with opportunities, challenges and limitations they present. The next session covered novel targets and discovery approaches that also pertain to enabling technologies – as these will be critical to enable adoptive T cell therapies against a broader range of targets with leaky or very narrow expression profiles, respectively. Last but not least, the final session covered approaches to characterize mechanistic aspects of adoptive T cell therapy in clinic, through detailed understanding of immune pharmacodynamics markers as a key pre-requisite to spearheading such technologies through development and to commercialization.

## Chimeric antigen receptors

The event was opened with a keynote presentation by Dr. Zelig Eshhar from the Weizmann Institute of Science, who pioneered the concept of T-bodies or CARs during the late 80’s and early 90’s.

In his presentation entitled “CAR T Cells from the Mouse Cage to the Patients’ Health”, Dr. Eshhar provided a brief chronicle description of the pioneering of the CAR strategy and its emergence and evolution for adoptive cell treatment of cancer. The presentation, primarily covered experimental models for cancer amenable to testing adoptive T cell therapies, and summarized the key lessons learned from such models. The potential and challenges for adoptive cell therapy in cancer patients were also discussed, along with current hurdles that need to be overcome.

Following Dr. Eshhar, in his presentation entitled “T Cell Therapy: Target Antigen Discovery and Clinical Translation”, Dr. Richard Morgan from Bluebird Bio emphasized the importance of tumor antigen discovery and careful selection of targets in CAR T cell therapy. To illustrate the challenges and opportunities afforded by current targets pursued in the context of CAR technologies, he compared and contrasted Her2/neu and EGFRvIII as solid tumor targets. Dr. Morgan also discussed elements related to clinical translation of these two CAR-based therapies and results to date.

Finally, B cell leukemias and lymphomas are primary targets for current CAR T cell products in development. However, some of the most pursued antigens such as CD19 and CD20, as lineage associated targets, are associated with on target/off tumor toxicities. More specifically, while T cells expressing chimeric antigen receptors (CARs) targeting B-cell malignancies show remarkable clinical efficacy, their long term persistence causes depletion of normal B cells and hypogammaglobulinemia because the antigens targeted do not discriminate tumor from normal B cells. Dr. Carlos Ramos from Baylor College of Medicine, in his presentation entitled “Clinical Responses in Patients Infused with T Lymphocytes Redirected to Target Κ-Light Immunoglobulin Chain” highlighted the potential benefits of targeting the immunoglobulin itself. Since B-cell malignancies express either a κ or a λ-light immunoglobulin they generated a CAR (κ.CAR) specific for κ-light chain to selectively target κ+ lymphoma/leukemia cells, while sparing the normal B cells expressing the non-targeted λ-light chain. Dr. Ramos presented results of a phase I clinical of T cells targeting this antigen.

### T cell receptor targets and products

T cell receptor (TCR)-engineered T cells represent a different targeting approach amenable to tapping into the internal proteome, which was the subject of the second session of this conference.

This session was opened by Dr. Achim Jungbluth, from Memorial Sloan-Kettering Cancer Center with a presentation entitled “Overview of the Protein Expression of CT Antigens”. Cancer Testis (CT) antigens, a major focus of cancer vaccine targets as well as TCR –engineered T cell products currently in development, were originally identified by their ability to elicit cellular and/or humoral autologous immune responses in tumor patients. As their name implies, classical CT antigens are typically present in various types of malignant tumors, while their expression in normal adult tissues is mainly restricted to testicular germ cells. Due to their tumor-associated expression pattern, they are considered appealing targets for vaccine-based immunotherapy of cancer. In spite of their identification almost 15 years ago and numerous studies, there is debate as to the protein expression of CT antigens in tumors. This is partially due to the serological typing reagents to CT antigens. The presentation addressed issues of proper immunohistochemical typing and typing reagents for classical CT antigens and an overview was given about what can be considered solid data about the protein expression of CT antigens in various types of tumors.

The next presentation discussed the concept of utilizing a soluble TCR-based targeting approach to re-direct a patient’s T cell repertoire against cells presenting targets of interest. Dr. Emma Hickman from Immunocore gave a presentation entitled “Target Discovery for TCR-Mediated Immunotherapy of Cancer with ImmTACs”. The presentation began with an introduction to ImmTACs and went on to describe Immunocore’s approach to target identification and target profiling for the ImmTAC platform. ImmTACs are bi-specific reagents that comprise an engineered pico-Molar affinity TCR fused to an anti-CD3 specific scFv. Through their high affinity target recognition and potent T cell re-direction, ImmTACs are able to mediate a T cell response against cancer cells that present low numbers of epitopes. Emerging clinical data for the most advanced ImmTAC reagent to-date, IMCgp100, indicates that the drug is well tolerated and can induce T cell mobilization and tumour shrinkage in patients with advanced melanoma. Although TCRs have access to a large pool of potential targets, careful analysis is required to confirm epitope presentation and to ensure disease specificity. Therefore, to complement the ImmTAC platform, a comprehensive validation pathway has been developed to identify and validate new targets. This process includes state of the art mass spectrometry for both de novo and targeted searching, and detailed target expression profiling. Through these investigations Immunocore has created an expanding database of fully-validated targets.

This session was closed by Dr. Joanna E. Brewer from Adaptimmune Ltd. with her presentation “Fine-Tuning TCR-Antigen Specificity and Predicting Potential Off-Target Reactivity”. Adoptive T cell therapy (ACT) with gene-modified T cells expressing exogenous TCRs is emerging as a highly promising strategy for the treatment of many types of cancer. Mutating T cell receptors, to improve their affinity for tumor specific epitopes, provides the needed potency for efficacy but may present safety concerns for off-target recognition as TCRs effectively bypass thymic selection. The species-specific nature of TCR-MHC interactions means that in vivo models are not suitable for identifying potential safety risks that could arise in patients. The use of peptide bioinformatics offers a robust delineation of any possible peptide epitopes that can be recognised by an individual TCR. TCRs isolated from different donors show different binding recognition patterns along the epitope and will have different but overlapping arrays of possible off target peptides. The bioinformatic information is complemented by work with transduced and primary cells to determine whether those peptides identified can be processed and presented on HLA. Many peptides that could be recognised by a TCR are not expressed at the cell surface and as such may not be considered to pose a risk to patient safety. Dr. Brewer discussed a systematic approach that was devised in response to off-target toxicity seen in the clinic, to optimize and de-risk novel TCRs for new treatments.

### New targets: from discovery to clinic

As the current list of targets pursued within the framework of adoptive T cell therapies is limited and presents challenges, a novel direction is the research of new targets, categories of targets and enabling technologies.

Dr. Paul F. Robbins from the National Cancer Institute opened this session with his presentation entitled “Developing Potent Cancer Therapies Targeting Cancer Germline and Mutated Antigens”. While current cancer gene therapy trials have focused on transducing autologous T cells with T cell receptors (TCRs) that recognize tumor antigens with limited or no normal tissues expression, some severe toxicities were observed in clinical trials targeting differentiation antigens. In a recent trial utilizing a TCR directed against the cancer germline antigen NY-ESO-1, whose expression is limited to the normal testis, objective clinical response rates of 58 and 56% were observed in patients with melanoma and synovial cell sarcoma, respectively, but no normal tissue toxicities attributed to the transferred T cells were observed. Additional strategies were also being developed to target mutated epitopes that are generally limited in their expression to a single patient but that may represent particularly potent targets for therapy. Dr. Robbins discussed this new paradigm of targeting mutated epitopes towards generating a new category of individualized TCR products.

While novel targets represent a key research direction in the field, there is the emerging realization that in order to fully tap into the adoptive T cell therapy technology, one needs to define and leverage methods to enable the activity of T cells *in vivo*, in light of many barriers associated for example with the tumor microenvironment.

In his presentation titled “In vivo Discovery of Targets for Cancer Immunotherapy”, Dr. Kai Wucherpfennig from Harvard Medical School presented a novel pooled shRNA screen that has been developed to discover genes that represent key negative regulators of cytotoxic T cell function in tumors. shRNAs that target such negative regulators greatly enhance T cell accumulation in tumors and improve T cell function. This approach enables systematic target discovery in relevant tissue microenvironments, and co-targeting of such pathways could prove essential for generating efficacious T cell products with long lasting clinical effect.

This session dedicated to new targets was closed by Dr. Stanley Frankel, from Amgen, with his presentation “Bridging T Cells to Tumors: Clinical Progress with BiTE Antibodies”. This platform technology aims to re-direct patient’s T cells against membrane borne target antigens *in vivo*, through the utilization of recombinant bispecific proteins that bridge antigen-expressing target cells with CD3+ T cells. Importantly, efforts aimed at discovering novel targets for BiTE antibodies could also yield viable targets for CAR T cell products. In his presentation, Dr. Frankel provided a perspective of where the BiTE pipeline currently is in terms of development, and potential next products and targets to enter development.

### Monitoring the performance of t cell products

In the last session of the conference we focused on approaches to characterize mechanistic aspects of adoptive T cell therapy in clinic, through detailed understanding of immune pharmacodynamics markers as a key pre-requisite to spearheading such technologies through development and to commercialization.

In his presentation entitled “Immune Profiling during Pre-Clinical Development and in Phase I/II Clinical Trials”, Dr. Yoav Peretz from Caprion, highlighted why immune monitoring of patients enrolled in clinical trials has become crucial to help guide the development of immunotherapies and reveal potential relationships between the clinical and immunological response. The lack of protective signatures is partly explained by the realization that monoparametric evaluations fail to capture the phenotypic and functional heterogeneity of the immune response. Multiparametric flow cytometry has become the tool of choice given its ability to simultaneously analyze the distribution of cellular phenotypes and functions in complex mixtures of cells such as in peripheral blood and tissues. In a study aimed at identifying correlates of hyporesponse in the elderly, Dr. Peretz showed that specific phenotypic signatures are associated with aging populations, partly explaining the immune hyporesponsive phenomena in the elderly. Overall, the use of these analytical tools to evaluate changes in the phenotype and function of cellular subsets during disease and after treatment enables the identification of correlates and predictive biomarkers of immune protection.

A key therapeutic modality within the landscape of adoptive T cell therapies is represented by tumor infiltrating lymphocytes (TILs). Dr. Laszlo Radvanyi, from Lion Biotechnologies closed the session and the conference with his presentation entitled “Emerging Biomarker Approaches to Predict Response in Melanoma T Cell Therapy”. The high clinical response rates (up to 50%) in melanoma patients receiving autologous tumor-infiltrating lymphocyte (TIL) therapy offer an unprecedented opportunity to identify both on-treatment and predictive immunotherapy biomarkers. Dr. Radvanyi presented work on a comprehensive systems biology platform identifying biomarkers of response and resistance in a diverse array of specimen types from patients receiving TIL therapy. A number of TIL phenotypic markers and genomic markers in tumors predictive of response and improved survival after therapy have been identified; these markers also beginning to shed light on the mechanisms underlying therapy resistance.

## Conclusions and directions

The conference discussed challenges and opportunities associated with current platform technologies for adoptive T cell therapy, mainly from the target discovery point of view. Several versions of T cell therapies were presented and discussed: CAR and TCR products against generic targets, bispecific recombinant proteins redirecting the activity of T cells against tumor targets (Immtacs and BiTEs). All these have great potential but a limiting factor is the number of currently available targets, as an optimal expression profile and other characteristics are desirable features. This drove immense interest in discovering new targets and novel classes of targets expressed on cell membrane or inside the cell, mainly tumor specific antigens and especially mutated antigens. In terms of future directions of research, along with advancing novel targets, enabling technologies are equally important. Among those, relevant stromal targets and approaches to reprogram the tumor environment and enable *in vivo* activity of adoptively transferred T cells are particularly important, along with strategies and assays to monitor the activity of T cells *in vivo*. As we are witnessing the first generation of adoptive T cell therapies in clinical development, there is a vast opportunity that can be tapped into through expanded effort to bring forward next generation technologies in this space targeting a broader range of molecules intimately associated with the cancer biology.

